# The effect of music on simulated surgical performance: a systematic review

**DOI:** 10.1007/s00464-019-06868-x

**Published:** 2019-05-28

**Authors:** Pim Oomens, Victor Xing Fu, Gert Jan Kleinrensink, Johannes Jeekel

**Affiliations:** 1 Department of Neuroscience, Erasmus MC, University Medical Center, Doctor Molewaterplein 40, 3015 Rotterdam, The Netherlands; 2 Department of Surgery, Erasmus MC, University Medical Center, Doctor Molewaterplein 40, 3015 Rotterdam, The Netherlands

**Keywords:** Music, Surgical performance, Surgery, Surgical skill, Surgical education, Laparoscopy

## Abstract

**Introduction:**

Beneficial effects of music have been described on several cognitive domains, task performance, stress, anxiety and pain. Greater surgical skill is a factor that has been associated with improved patient outcome. The aim of this systematic review is to assess the effect of music on surgical performance.

**Methods:**

An exhaustive literature search was performed. The following databases were searched: Embase, Medline Ovid, Web of Science, Cochrane CENTAL, PsycINFO Ovid, CINAHL EBSCOhost, ERIC EBSCOhost and Google Scholar. All prospective studies that assessed the effect of a music intervention compared to either another auditory condition or silence on surgical performance were included in a qualitative synthesis. The study was registered in the PROSPERO-database (CRD42018092021).

**Results:**

The literature search identified 3492 articles of which 9 studies (212 participants) were included. Beneficial effects of music were reported on time to task completion, instrument handling, quality of surgical task performance and general surgical performance. Furthermore, a beneficial effect of music on muscle activation was observed.

**Conclusion:**

Although beneficial effects of music on surgical performance have been observed, there is insufficient evidence to definitively conclude that music has a beneficial effect on surgical performance in the simulated setting. Future studies should be conducted using greater numbers of participants focusing on a more limited range of tasks, as well as validation in the live operating environment.

Music is played during surgery in many operating rooms (ORs) worldwide. A majority of physicians and nurses reported that they listen to music on a regular basis in the OR [1, 2]. Respondents stated that music makes them feel calmer and work more efficient. Rauscher et al. first described beneficial effects of music on spatial task performance [3]. Since then, much has been published on this so-called Mozart effect. A meta-analysis concluded that there is a small but statistically significant beneficial effect of listening to Mozart on task performance. Moreover, this effect can also be observed with other types of music [4]. Beneficial effects of music have been reported on task performance and cognitive abilities in both rodents and humans [5–8]. Furthermore, anxiolytic and analgesic effects of music during surgery have been observed [9]. Also, stress-reductive effects of music in healthcare professionals have been described [10].

Greater surgical skill has been associated with a reduction in postoperative complications [11] and high stress levels in the operating theatre can negatively affect surgical performance and team performance [12]. According to a survey, nearly 80% of the responding surgeons experience pain on a regular basis while performing surgery [13]. Since music can improve task performance, reduce stress and has analgesic effects, it could potentially benefit surgical performance and therefore patient outcome. The primary objective of this systematic review is to assess the effect of music on surgical performance. Secondary outcomes are the effect of music on vital parameters, stress and electromyography (EMG).

## Methods

The study protocol was registered in the PROSPERO database (CRD42018092021) [14]. All aspects of the PRISMA-statement were followed [15]. Neither IRB approval nor written informed was necessary to obtain, as this paper is a systematic review.

### Search strategy

The databases Embase, Medline Ovid, Web of Science, Cochrane CENTAL, PsycINFO Ovid, CINAHL EBSCOhost, ERIC EBSCOhost and Google Scholar were searched on 1 March 2018 with keywords like “surgery” “surgical skill” “music” and “auditory stimulation”. The syntax construction and database search were executed in collaboration with a biomedical information specialist using the exhaustive search method [16]. The full search and syntax is presented in Appendix. Two independent reviewers (PO and VF) identified eligible studies. First, all identified articles were screened by title and abstract. Subsequently, the full text articles were screened to assess if eligibility criteria were matched. Only full text peer-reviewed published articles in the English language were included. Inclusion criteria for this systematic review were prospective studies that assessed the effect of music compared to another auditory condition or to silence on surgical performance. Secondary outcomes were the effect of music on heart rate, blood pressure, stress response and electromyography (EMG). Studies were excluded if multiple concomitant interventions were used. Discrepancies were resolved through mutual discussion or by referring to a senior author (JJ).

### Data collection and quality assessment

Data collection was performed independently by two researchers (PO and VF) using customised forms. If data were available in plots or images, data were estimated using the online available data extraction software WebPlotDigitizer (version 4.1) [17]. If necessary, authors were contacted to obtain additional data. Risk of bias was assessed using the Cochrane Collaboration’s tool for assessing risk of bias [18]. Disagreements between reviewers were resolved through mutual discussion or by referring to a senior author (JJ).

### Data analysis

The overall group path length and time to task completion (TTC) means were calculated if individual data were presented. Standard error was converted to standard deviation as described in the Cochrane handbook [18]. If a study contained several music interventions, the means and standard deviations of the different music groups were pooled to an approximated mean and standard deviation of the entire group. If several tasks were used to assess surgical performance, approximated means and standard deviations were pooled for the outcomes of time to task completion and path length. If absolute means were presented, mean differences and percentages of mean differences were computed. Only the percentage of improvement was extracted in studies where the task that was used in the intervention group was different from the task in the control group, as parameters such as time to task completion and path length inherently differ between the different tasks.

## Results

**Fig. 1:**
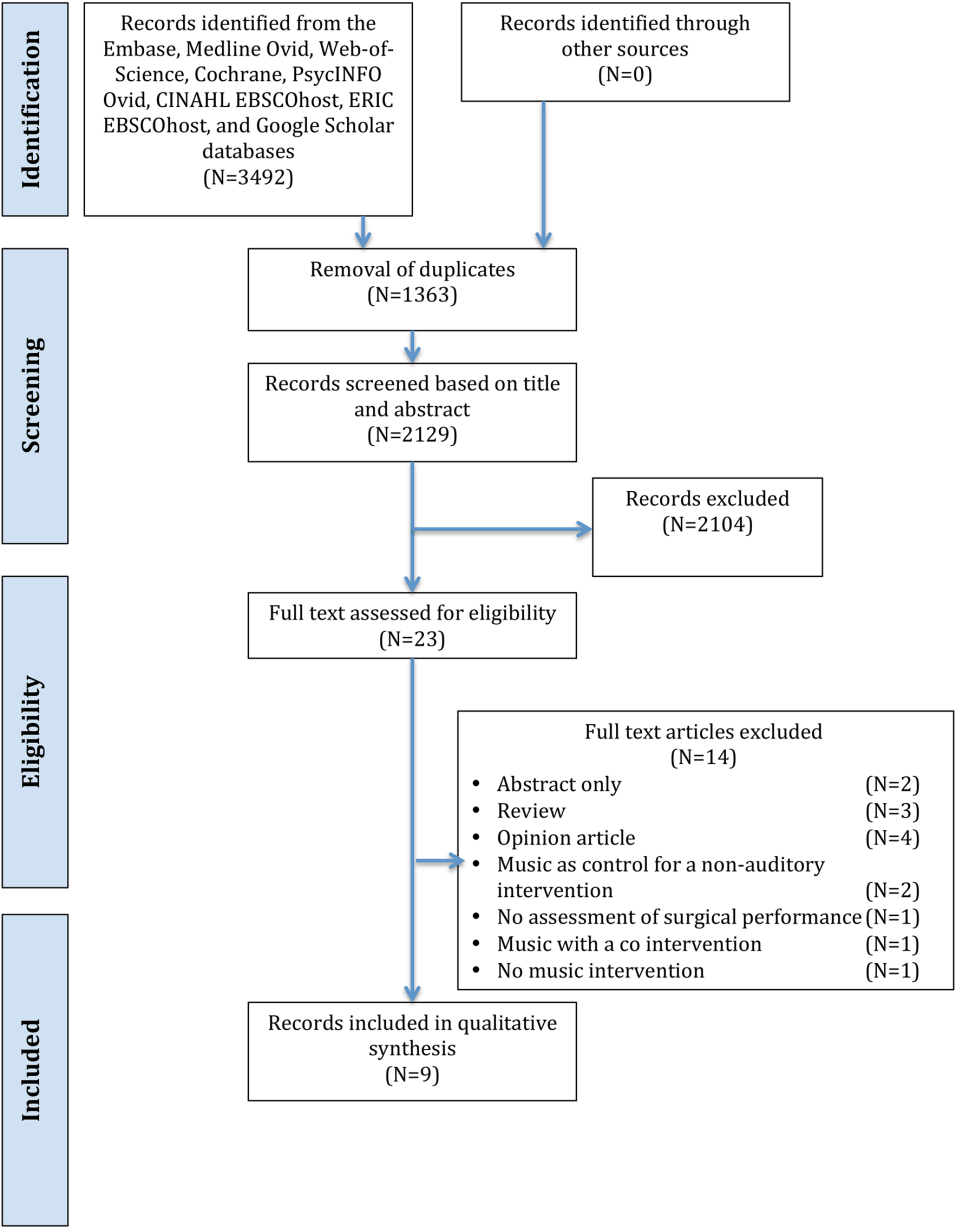
PRISMA flow diagram. N = number of articles

The PRISMA flow diagram of the search strategy is presented in Fig. 1. Initial database searching resulted in 3492 articles (2129 after removal of duplicates). Nine articles (212 participants) were included in this review. An overview of the study characteristics is presented in Table 1. All studies assessed surgical performance in a simulated setting. In eight studies, the music intervention was applied during the assessment of the surgical performance [19–26]. One study applied the music intervention prior to performing the simulation tasks [27]. Motion analysis software was used to assess surgical performance in six studies [19–21, 23–26].

**Table 1:** Study characteristics of the included studies

Study	Study design	Participants	Technique	Music intervention	Control intervention	Simulation used	Performance assessment
Conrad [19]	Crossover	8 Experienced surgeons	Laparoscopy	Classical (Mozart piano sonatas)	Silence Dichotic music^a^ Mental loading	Surgical SIM VR ∙ Tasks not specified	Time to task completion Path length
Conrad [20]	Crossover	31 Junior surgeons^b^	Laparoscopy	Classical (Mozart piano sonatas)	Silence Dichotic music^a^ Mental loading	Surgical SIM VR ∙ Lifting a structure and cutting ∙ Targeting objects ∙ Feeding a rope ∙ Aligning objects	Time to task completion Path length
Kyrillos [21]	Crossover	12 Residents 14 Ophtalmologists	Intraocular surgery	Classical (Mozart KV 448)	No music	EyeSI VRmagic ∙ CAT-A Anti tremor task ∙ CAT-C Capsulorhexis	Time to task completion Total score Percentage out of bound Deviation from radius Roundness Centering
Lies [22]	Crossover	12 Residents	Open surgery	Preferred music	No music	Pig’s foot ∙ Layered wound closure	Time to task completion Quality
Miskovic [23]	Randomized controlled trial	45 Junior surgeons^b^	Laparoscopy	Activating music Deactivating music	No music	Xitact LS500 ∙ Clipping and cutting cystic duct	Time to task completion Path length Global score
Moorthy [24]	Crossover	12 Experienced surgeons	Laparoscopy	Classical	OR-noise No music	Pelvitrainer ∙ Placing of a suture	Time to task completion Path length Global rating scale Accuracy count Knot quality
Shakir [25]	Crossover	9 Residents 3 Fellows	Microsurgery	Preferred music	No music	Chicken’s foot ∙ Arterial anastomosis	Motion analysis score
Siu [26]	Crossover	10 Medical students	Robotic surgery	Classical Hiphop Jamaican Jazz	No music	daVinci Skills Simulator ∙ Tying three knots ∙ Mesh alignment	Time to task completion Path length
Wiseman [27]	Crossover+ Cohort	55 Novices	Laparoscopy	Classical (Mozart KV 448) Progressive Metal (Dream Theater – Stream of Consciousness)	No music	Custom laparoscopic box ∙ Peg transfer ∙ Rope transfer ∙ Removal of a pen cap	Time to task completion Error score

^a^German Folk music in one ear, deathmetal music in the other ear

^b^Residents without prior laparoscopic experience

Classical music was used as a music intervention in six studies, while preferred music of the participant was used in two studies. All studies used silence or ‘no music’ as a control intervention. Additional auditory intervention groups consisted of dichotic music [19, 20], defined as two different types of music applied through each ear, and OR noise [24].

### Bias assessment

**Fig. 2:**
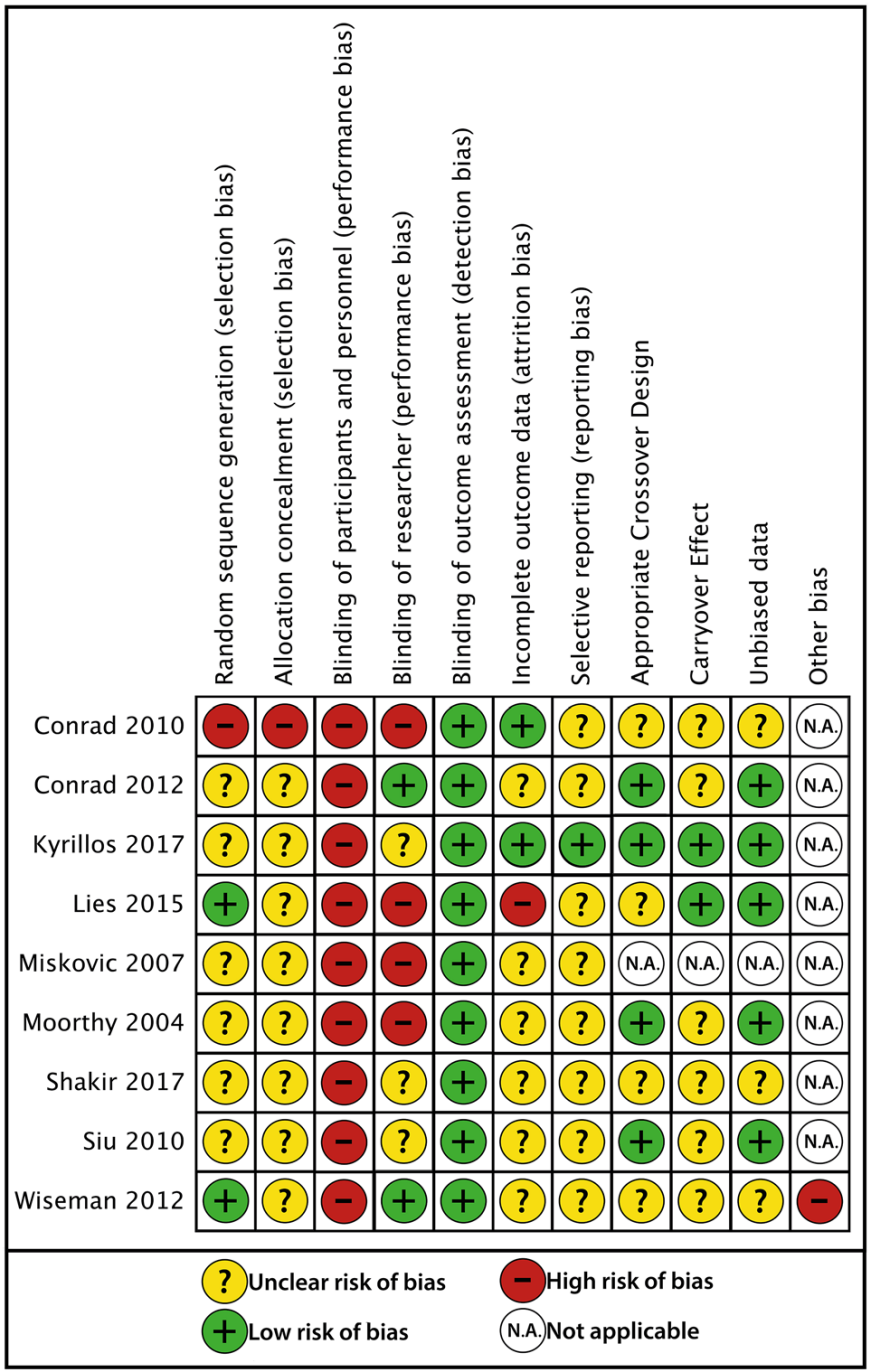
Risk of bias summary. Review authors’ judgements about each risk of bias item for each included study

**Fig. 3:**
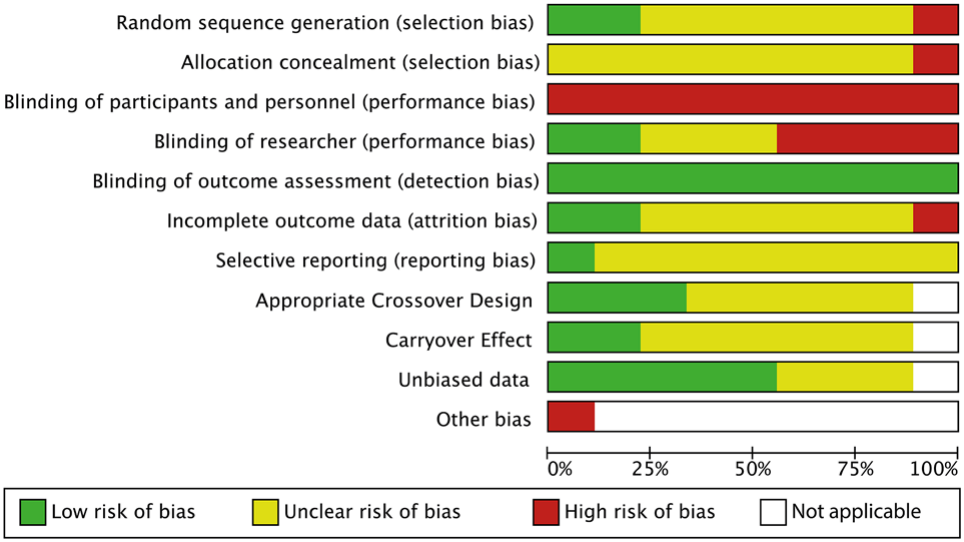
Risk of bias graph: Review authors’ judgements about each risk of bias item presented as percentages across all included studies

Risks of bias of the included studies are presented in Figs. 2, 3. Several studies lacked information to adequately assess all quality domains. Participants could inherently not be blinded due to the nature of the intervention; therefore, risk of performance bias was high in all studies. Detection bias was low in all studies since either motion analysis software or predefined criteria by blinded observers were used to assess surgical performance. The condition (i.e. surgical task performance) was considered to be suitable for a crossover study if subjects were allowed to practice the task first, or if subjects were experienced with the type of task that was performed, or if a learning effect was assessed and was absent. Carryover effect was assessed as low risk of bias in one study as the time between periods was at least 24 h with a median time of 15.5 days. All other crossover studies did not specify the washout period and carryover effect was therefore assessed as unclear risk of bias in these studies.

Other bias has been assessed in one study as high risk of bias, since the study design was changed during the experiment [27]. In this study, two music interventions were compared in a crossover study. An additional cohort with no music was added after analysis of the two music interventions.

### Effects of music on time to task completion

Eight studies assessed the effect of music on time to task completion (Table 2) [19–24, 26, 27]. Three studies evaluated whether the relative improvement in time to task completion was higher, when participants repeated a task and were exposed to either music, no music, silence or another auditory intervention [19, 20, 27]. In the study by Wiseman et al., each participant completed a series of three tasks [27]. The music cohort was exposed to classical music or progressive metal music during the second and third task, while the control cohort was not exposed to music. The percentage of improvement was not significantly different between the music groups and control group. In two studies by Conrad et al. with a similar setup, classical music was played throughout the entire experiment during both the first and second task. One of the two studies showed a statistically significant higher percentage of improvement when participants listened to music compared to the improvement measured during silence [20]. The other study did not report a level of significance as the study consisted of only eight participants [19].

**Table 2:** Effects of music on time to task completion (TTC)

Study	Intervention		Measure	Results		Mean difference
	Music	Control		Music	Control	
Assessment of improvement of TTC						
Conrad [19]	Mozart piano sonatas	Silence	Mean percentage of improvement	10.51	26.06	15,55
Conrad [20]	Mozart piano sonatas	Silence	Median percentage of improvement	61.29^c^	39.27	Not applicable
Wiseman [27]	Pooled music groups^a^ (n = 39)	No music (n = 15)	Mean percentage of improvement	22.78 ± 22.93	20.4 ± 9.1	-2.38

Study	Intervention		Measure	Results		Mean difference (% of mean difference)
	Music	Control		Music	Control	
Assessment of absolute TTC						
Kyrillos [21]	Mozart KV 448	No music	Mean TTC(sec.)	81.97	81.79	0.18 (-0.2%)
Lies [22]	Preferred music	No music	Mean TTC(min.) ± SD	10.6 ± 2.6*	11.5 ± 2.9	-0.9 (7.8%)
Miskovic [23]	Activating music	No music	Mean TTC	Not specified		Not applicable
	Deactivating music					
Moorthy [24]	Classical	Silence	Median TTC(sec.) (IQR)	529 (210.8)	585.5 (149.6)	Not applicable
		OR-noise			526.8 (275.1)	
Siu [26]	Pooled music groups^b^	No music	Mean TTC(sec.) ± SD	70.36 ± 29.25^d^	88.56 ± 31.09	-18.20 (20.6%)

If the number of participants is not equally divided between groups, the number of participants is specified between parentheses in the intervention columns

*TTC* time to task completion

^a^Mozart KV448 and Dream Theater-Stream of Consciousness

^b^Classical, hiphop, Jamaican, and Jazz

^c^p < 0.05

^d^p < 0.05 in both hiphop and Jamaican music compared to control

Four studies evaluated the mean time to task completion with and without music [21, 22, 24, 26]. Two studies reported a statistically beneficial effects of both preferred music, hiphop and Jamaican music, on time to task completion [22, 26]. One study did not present exact values, but reported no significant difference between groups [23].

### Effects of music on instrument handling

Instrument handling, defined as path length (i.e. the total distance travelled by the instrument tip) or as the percentage of time that the instrument was out of a predefined boundary, was assessed in six studies (Table 3) [19–21, 23, 24, 26].

**Table 3:** Effects of music on instrument handling

Study ID	Interventions		Parameter of assessment	Measure	Results		Mean difference
	Music	Control			Music	Control	
Assessment of improvement of instrument handling							
Conrad [19]	Mozart piano sonatas	Silence	Path length^b^	Mean percentage of improvement	–5.29	21.56	–26,85
Conrad [20]	Mozart piano sonatas	Silence	Path length^b^	Median percentage of improvement^f^	57.00^d^	32.02	Not applicable

Study ID	Interventions		Parameter of assessment	Measure	Results		Mean difference (% of mean difference)
	Music	Control			Music	Control	
Assessment of instrument handling							
Kyrillos [21]	Mozart KV. 448	No music	Percentage out of bound^c^	Mean (percentage)	18.83	20.24	–1,68 (8,3%)
Moorthy [24]	Classical	Silence	Path length^b^	Median (cm) (IQR)	7124.4 (15,809.4)	6608.2 (3233.5)	Not applicable
		OR-noise				7192.2 (5732.9)	
Miskovic [23]	Activating music	No music	Path length^b^	Not specified	Not specified		Not applicable
	Deactivating music						
Siu [26]	Pooled music groups^a^	No music	Path length^b^	Mean (cm) ± SD	1130.81 ± 414.80^e^	1398.26 ± 417.97	-267,45 (19,13%)

^a^Classical, hiphop, Jamaican and Jazz

^b^The total distance travelled by the instrument tip

^c^The percentage of time the instrument was out of a predefined boundary

^d^p < 0.05

^e^p < 0.05 in Jamaican music compared to control

Two studies with a similar setup assessed whether improvement of path length was higher when participants repeated a task and were exposed to either classical music, no music, another auditory intervention or silence [19, 20]. One study found that improvement of path length upon repetition was statistically significantly increased during exposure to classical music in comparison to any other control condition [20]. The other study did not report a level of statistical significance, as only eight participants were included [19].

Four studies evaluated the mean path length or percentage out of bound with and without exposure to music. A statistically significant beneficial effect of Jamaican music was found on path length in the study conducted by Siu et al. [26.]. One study did not present exact values, but reported no significant difference between groups [23].

### Effects of music on surgical task performance quality

The quality of the performed surgical task was assessed in two studies by blinded observers using predefined criteria [22, 24]. Wound, repair graded on a 1–5 scale by blinded plastic surgeons, was performed with significantly better quality when participants listened to their preferred music genre [22]. There was no statistically significant effect of classical music on the quality of a laparoscopically tied knot [24].

### Effects of music on general surgical task performance

Four studies assessed the effect of music on a general score rating surgical task performance [21, 23–25]. Two studies used the total score, generated by the simulator’s built-in software [21, 23]. One study used a validated global rating scale developed by Reznick et al. [24, 28.]. Shakir et al. used a validated general motion analysis score based on the parameters time to task completion, tremor, extreme movements and overall movement pattern. This general motion analysis score was significantly improved during exposure to preferred music [25]. Significant beneficial effect of classical music on the total score was also observed in simulated intra-ocular surgery [21]. Two studies did not find a statistically significant effect of classical music, activating or deactivating music on the total score [23, 24].

### Effect of music on vital parameters and muscle activation

One study assessed the effect of music on heart rate and heart rate variability (HRV) during surgical performance [23]. Listening to activating music during surgical performance led to an increased heart rate compared to deactivating music and ‘no music’. There were no significant differences in HRV.

One study assessed the effects of music on muscle activation in the dominant hand using electromyography (EMG) as an indication of muscle fatigue [26]. Mean electromyography activation of the extensor digitorum muscle was significantly reduced when participants listened to any type of researcher-selected music (i.e. classical, hiphop, Jamaican or jazz), while median electromyography frequency did not differ statistically significantly between groups. Music did not have a statistically significant effect on mean electromyography activation of the flexor carpi radialis, but did decrease median electromyography frequency.

## Discussion

This systematic review provides an overview of the effect of music on surgical performance. Five out of nine studies reported beneficial effects of music on different surgical performance domains. Beneficial effects of music were observed on TTC [20, 22, 26], instrument handling [20, 22, 26], task performance quality [22] and general surgical task performance [21, 25]. Moreover, one study also observed an attenuating effect of music on muscle activation, which can be correlated to muscle fatigue [26, 29].

All included studies assessed the effect of music on surgical skill in a simulated setting. Surgical skill acquired in a simulated setting translates to and correlates with surgical performance in a clinical setting [30–35]. Greater surgical skill is associated with a lower mortality and complication rate in surgical patients, including surgical site infections, pulmonary complications, readmissions and reoperations [11]. Several studies reported a beneficial effect of music on time to task completion. Prolonged operation duration has been associated with a higher postoperative complication rate and increases medical costs [36, 37]. Therefore, the use of music during surgical procedures could potentially improve patient outcome and reduce costs, as one minute of OR-time is estimated to cost $36-37 [37, 38]. Implementing music interventions in training modules might also benefit residents. Simulation based training is an essential part of surgical education, as the American Board of Surgery Graduating requires graduating residents to successfully pass the FLS program (Fundamentals of Laparoscopic Surgery) [39, 40].

The type of music that is most beneficial is unclear, but we believe it to be unlikely that a surgeon would listen to music that they dislike. Perhaps the beneficial effect of music on surgical performance is more profound if participants can choose music of their preference. This would coincide with earlier observations where the beneficial effect of music on the surgeon’s physiological response was larger under self-selected music compared to researcher-selected music [41]. Out of the nine included studies in this review, two used preferred music of the participants. Both these studies observed statistically significant beneficial effects of music on time to task completion, task performance, quality of repair and on general surgical task performance [22, 25]. Siu et al. used several researcher-selected music genres. Significant beneficial effects of hiphop were observed on time to task completion, hiphop was in the top two favourite genres of 70% of the participants [26]. In another study, a tendency towards improved surgical performance was observed in participants that rated the music as pleasant, compared to unpleasant or to silence [23].

There are several limitations of this review. One limitation is the low number of included studies and participants. While time to task completion was assessed as the primary outcome measure by most studies, it was not reported in a consistent manner. Some studies reported within-subject improvement, while others reported absolute means of the groups. Moreover, the studies contained different simulated tasks. Therefore, no meta-analysis could be performed, and no absolute values (i.e. time reduction in minutes) could be calculated. Other endpoints were reported less frequently. This limits the strength of conclusions that could be drawn.

None of the included studies were performed in a live operating environment. There is contradicting evidence with regard to the use of music in the operating theatre. Music has been reported to reduce stress and increase working efficiency in OR-staff [1, 2]. Music has also been reported to impair surgeon’s auditory processing and team communication [44, 45]. The majority of anaesthetists generally like music in the operating theatre, but also consider it to be distracting if anaesthesiological problems were to occur [42]. However, in a simulated setting, no adverse effects of music were observed on anaesthetist’s psychomotor performance [43]. Many factors can potentially affect surgical performance in a live operating environment, including leadership skills, communication level and cooperation [46–50]. How music affects all these factors and thus surgical performance in a live operating environment is unclear. Nonetheless, several studies have reported a correlation between improved surgical performance in a simulated setting and performance in the live operating environment [30–35].

## Conclusion

There is no sufficient evidence to definitively determine whether music has a beneficial effect on surgical performance in the simulated setting. However, the results suggest that preferred music of the participant does improve surgical performance in a simulated setting. Future studies should be conducted using greater numbers of participants, participant preferred music, and focusing on a more limited range of tasks. Furthermore the effects of music on surgical team performance and patient outcome should be assessed, in order to answer the question whether music improves surgical performance in the live operating environment.

## Appendix

### Search strategy

#### embase.com

(music/de OR ‘auditory stimulation’/de OR ‘noise’/de OR (music OR musical OR musicotherap* OR (rhythm NEAR/3 (perception* OR accompan*)) OR melod* OR ((auditor* OR acoustic*) NEAR/3 (distract* OR condition* OR stress* OR relax* OR stimulat*)) OR noise):ab,ti) AND (‘surgical skill’/exp OR ‘suture’/de OR ‘wound closure’/de OR ‘suture technique’/de OR ‘surgeon’/exp OR ((‘motor system’/de OR ‘psychomotor performance’/de OR ‘motor performance’/de OR ‘motor function test’/de OR ‘task performance’/de OR ‘eye hand coordination’/de OR ‘motor activity’/de OR ‘motor coordination’/de) AND (surgery/exp OR ‘operating room’/exp)) OR (((motor* OR psychomotor* OR performan* OR abilit* OR function* OR skill* OR train* OR entrain* OR education* OR learn* OR simulat* OR improv* OR sequence* OR process* OR interaction* OR coordinat* OR task*) NEAR/3 (surgic* OR surger* OR operating-room* OR operating-theat* OR laparoscop* OR perioperat* OR peroperat* OR peri-operat* OR per-operat*)) OR surgeon* OR stitch* OR sutur* OR laparoscop* OR davinci OR da-vinci):ab,ti) NOT ([animals]/lim NOT [humans]/lim) AND [english]/lim.

#### Medline Ovid

(music/OR Acoustic Stimulation/OR noise/OR (music OR musical OR musicotherap* OR (rhythm ADJ3 (perception* OR accompan*)) OR melod* OR ((auditor* OR acoustic*) ADJ3 (distract* OR condition* OR stress* OR relax* OR stimulat*)) OR noise).ab,ti.) AND (sutures/OR Suture Techniques/OR Wound Closure Techniques/OR exp surgeons/OR ((“Task Performance and Analysis”/OR Psychomotor Performance/OR motor activity/) AND (exp Surgical Procedures, Operative/OR Operating Rooms/)) OR (((motor* OR psychomotor* OR performan* OR abilit* OR function* OR skill* OR train* OR entrain* OR education* OR learn* OR simulat* OR improv* OR sequence* OR process* OR interaction* OR coordinat* OR task*) ADJ3 (surgic* OR surger* OR operating-room* OR operating-theat* OR laparoscop* OR perioperat* OR peroperat* OR peri-operat* OR per-operat*)) OR surgeon* OR stitch* OR sutur* OR laparoscop* OR davinci OR da-vinci).ab,ti.) NOT (exp animals/NOT humans/) AND english.la.

#### PsycINFO Ovid

(music/OR Auditory Stimulation/OR noise effects/OR (music OR musical OR musicotherap* OR (rhythm ADJ3 (perception* OR accompan*)) OR melod* OR ((auditor* OR acoustic*) ADJ3 (distract* OR condition* OR stress* OR relax* OR stimulat*)) OR noise).ab,ti.) AND (exp surgeons/OR (((motor* OR psychomotor* OR performan* OR abilit* OR function* OR skill* OR train* OR entrain* OR education* OR learn* OR simulat* OR improv* OR sequence* OR process* OR interaction* OR coordinat* OR task*) ADJ3 (surgic* OR surger* OR operating-room* OR operating-theat* OR laparoscop* OR perioperat* OR peroperat* OR peri-operat* OR per-operat*)) OR surgeon* OR stitch* OR sutur* OR laparoscop* OR davinci OR da-vinci).ab,ti.) NOT (exp animals/NOT humans/) AND english.la.

#### CINAHL EBSCOhost

(MH music OR MH Acoustic Stimulation OR MH noise OR TI (music OR musical OR musicotherap* OR (rhythm N2 (perception* OR accompan*)) OR melod* OR ((auditor* OR acoustic*) N2 (distract* OR condition* OR stress* OR relax* OR stimulat*)) OR noise) OR AB (music OR musical OR musicotherap* OR (rhythm N2 (perception* OR accompan*)) OR melod* OR ((auditor* OR acoustic*) N2 (distract* OR condition* OR stress* OR relax* OR stimulat*)) OR noise)) AND (MH sutures OR MH Suture Techniques OR MH surgeons + OR ((MH “Task Performance and Analysis” OR MH Psychomotor Performance + OR MH motor activity) AND (MH Operating Rooms OR MH Surgery, Operative +)) OR TI (((motor* OR psychomotor* OR performan* OR abilit* OR function* OR skill* OR train* OR entrain* OR education* OR learn* OR simulat* OR improv* OR sequence* OR process* OR interaction* OR coordinat* OR task*) N2 (surgic* OR surger* OR operating-room* OR operating-theat* OR laparoscop* OR perioperat* OR peroperat* OR peri-operat* OR per-operat*)) OR surgeon* OR stitch* OR sutur* OR laparoscop* OR davinci OR da-vinci) OR AB (((motor* OR psychomotor* OR performan* OR abilit* OR function* OR skill* OR train* OR entrain* OR education* OR learn* OR simulat* OR improv* OR sequence* OR process* OR interaction* OR coordinat* OR task*) N2 (surgic* OR surger* OR operating-room* OR operating-theat* OR laparoscop* OR perioperat* OR peroperat* OR peri-operat* OR per-operat*)) OR surgeon* OR stitch* OR sutur* OR laparoscop* OR davinci OR da-vinci)) NOT (MH animals + NOT humans +) AND LA (english).

#### ERIC EBSCOhost

(MH music OR TI (music OR musical OR musicotherap* OR (rhythm N2 (perception* OR accompan*)) OR melod* OR ((auditor* OR acoustic*) N2 (distract* OR condition* OR stress* OR relax* OR stimulat*)) OR noise) OR AB (music OR musical OR musicotherap* OR (rhythm N2 (perception* OR accompan*)) OR melod* OR ((auditor* OR acoustic*) N2 (distract* OR condition* OR stress* OR relax* OR stimulat*)) OR noise)) AND (TI (((motor* OR psychomotor* OR performan* OR abilit* OR function* OR skill* OR train* OR entrain* OR education* OR learn* OR simulat* OR improv* OR sequence* OR process* OR interaction* OR coordinat* OR task*) N2 (surgic* OR surger* OR operating-room* OR operating-theat* OR laparoscop* OR perioperat* OR peroperat* OR peri-operat* OR per-operat*)) OR surgeon* OR stitch* OR sutur* OR laparoscop* OR davinci OR da-vinci) OR AB (((motor* OR psychomotor* OR performan* OR abilit* OR function* OR skill* OR train* OR entrain* OR education* OR learn* OR simulat* OR improv* OR sequence* OR process* OR interaction* OR coordinat* OR task*) N2 (surgic* OR surger* OR operating-room* OR operating-theat* OR laparoscop* OR perioperat* OR peroperat* OR peri-operat* OR per-operat*)) OR surgeon* OR stitch* OR sutur* OR laparoscop* OR davinci OR da-vinci)) NOT (MH animals + NOT humans +) AND LA (english).

#### Cochrane CENTRAL

((music OR musical OR musicotherap* OR (rhythm NEAR/3 (perception* OR accompan*)) OR melod* OR ((auditor* OR acoustic*) NEAR/3 (distract* OR condition* OR stress* OR relax* OR stimulat*)) OR noise):ab,ti) AND ((((motor* OR psychomotor* OR performan* OR abilit* OR function* OR skill* OR train* OR entrain* OR education* OR learn* OR simulat* OR improv* OR sequence* OR process* OR interaction* OR coordinat* OR task*) NEAR/3 (surgic* OR surger* OR operating-room* OR operating-theat* OR laparoscop* OR perioperat* OR peroperat* OR peri-operat* OR per-operat*)) OR surgeon* OR stitch* OR sutur* OR laparoscop* OR davinci OR da-vinci):ab,ti).

#### Web of science

TS = (((music OR musical OR musicotherap* OR (rhythm NEAR/2 (perception* OR accompan*)) OR melod* OR ((auditor* OR acoustic*) NEAR/2 (distract* OR condition* OR stress* OR relax* OR stimulat*)) OR noise)) AND ((((motor* OR psychomotor* OR performan* OR abilit* OR function* OR skill* OR train* OR entrain* OR education* OR learn* OR simulat* OR improv* OR sequence* OR process* OR interaction* OR coordinat* OR task*) NEAR/2 (surgic* OR surger* OR operating-room* OR operating-theat* OR laparoscop* OR perioperat* OR peroperat* OR peri-operat* OR per-operat*)) OR surgeon* OR stitch* OR sutur* OR laparoscop* OR davinci OR da-vinci))) AND LA = (english).

#### Google scholar

music|musical||”auditory|acoustic distraction|stress|relaxation”|noise surgeon|”surgical skills|tasks”.

## Abbreviations

EMG: Electromyography
FLS: Fundamentals of laparoscopic surgery
HRV: Heart rate variability
OR: Operating room or operating theatre
PRISMA: Preferred reporting items for systematic review and meta analyses
TTC: Time to task completion

## References

[1] Makama JG, Ameh EA, Eguma SA (2010). Music in the operating theatre: opinions of staff and patients of a Nigerian teaching hospital. Afr Health Sci.

[2] Ullmann Y, Fodor L, Schwarzberg I, Carmi N, Ullmann A, Ramon Y (2008). The sounds of music in the operating room. Injury.

[3] Rauscher FH, Shaw GL, Ky KN (1993). Music and spatial task performance. Nature.

[4] Pietschnig J, Voracek M, Formann AK (2010). Mozart effect-Shmozart effect: a meta-analysis. Intelligence.

[5] Mammarella N, Fairfield B, Cornoldi C (2007). Does music enhance cognitive performance in healthy older adults?The Vivaldi effect. Aging Clin Exp Res.

[6] Schellenberg EG (2005). Music and cognitive abilities. Curr Dir Psychol Sci.

[7] Schellenberg EG, Nakata T, Hunter PG (2007). Exposure to music and cognitive performance: tests of children and adults. Psychol Music.

[8] Xing Y (2016). Mozart, mozart rhythm and retrograde mozart effects: evidences from behaviours and neurobiology bases. Sci Rep.

[9] Kuhlmann AYR, de Rooij A, Kroese LF, van Dijk M, Hunink MGM, Jeekel J (2018). Meta-analysis evaluating music interventions for anxiety and pain in surgery. Br J Surg.

[10] Lai HL, Li YM (2011). The effect of music on biochemical markers and self-perceived stress among first-line nurses: a randomized controlled crossover trial. J Adv Nurs.

[11] Birkmeyer JD (2013). Surgical skill and complication rates after bariatric surgery. N Engl J Med.

[12] Chrouser KL, Xu J, Hallbeck S, Weinger MB, Partin MR (2018). The influence of stress responses on surgical performance and outcomes: Literature review and the development of the surgical stress effects (SSE) framework. Am J Surg.

[13] Soueid A, Oudit D, Thiagarajah S, Laitung G (2010). The pain of surgery: pain experienced by surgeons while operating. Int J Surg.

[14] The effects of music on surgical performance: a systematic review (2018) http://www.crd.york.ac.uk/PROSPERO/display_record.php?ID=CRD42018092021

[15] Liberati A (2009). The PRISMA statement for reporting systematic reviews and meta-analyses of studies that evaluate health care interventions: explanation and elaboration. PLoS Med.

[16] Bramer WM, Rethlefsen ML, Mast F, Kleijnen J (2017). Evaluation of a new method for librarian-mediated literature searches for systematic reviews. Res Synth Methods.

[17] Rohatgi A (2018) WebPlotDigitizer, Version 4.1 edn.,

[18] Higgins JPT, Green S (2011) Cochrane Handbook for Systematic Reviews of Interventions Version 5.1.0 [updated March 2011]. The Cochrane Collaboration,,

[19] Conrad C (2010). The effect of defined auditory conditions versus mental loading on the laparoscopic motor skill performance of experts. Surg Endosc Interv Tech.

[20] Conrad C (2012). A quality improvement study on avoidable stressors and countermeasures affecting surgical motor performance and learning. Ann Surg.

[21] Kyrillos R, Caissie M (2017). Effect of music on surgical skill during simulated intraocular surgery. Can J Ophthalmol.

[22] Lies SR, Zhang AY (2015). Prospective randomized study of the effect of music on the efficiency of surgical closures. Aesthet Surg J.

[23] Miskovic D, Rosenthal R, Zingg U, Oertli D, Metzger U, Jancke L (2008). Randomized controlled trial investigating the effect of music on the virtual reality laparoscopic learning performance of novice surgeons. Surg Endosc Interv Tech.

[24] Moorthy K, Munz Y, Undre S, Darzi A (2004). Objective evaluation of the effect of noise on the performance of a complex laparoscopic task. Surgery.

[25] Shakir A, Chattopadhyay A, Paek LS, McGoldrick RB, Chetta MD, Hui K, Lee GK (2017). The effects of music on microsurgical technique and performance: a motion analysis study. Ann Plast Surg.

[26] Siu KC, Suh IH, Mukherjee M, Oleynikov D, Stergiou N (2010). The effect of music on robot-assisted laparoscopic surgical performance. Surg Innov.

[27] Wiseman MC (2013). The mozart effect on task performance in a laparoscopic surgical simulator. Surg Innov.

[28] Reznick R, Regehr G, MacRae H, Martin J, McCulloch W (1997). Testing technical skill via an innovative “bench station” examination. Am J Surg.

[29] Al-Mulla MR, Sepulveda F, Colley M (2011). A review of non-invasive techniques to detect and predict localised muscle fatigue. Sensors (Basel).

[30] Nagendran M, Gurusamy KS, Aggarwal R, Loizidou M, Davidson BR (2013). Virtual reality training for surgical trainees in laparoscopic surgery. Cochr Database Syst Rev.

[31] Nagendran M, Toon CD, Davidson BR, Gurusamy KS (2014). Laparoscopic surgical box model training for surgical trainees with no prior laparoscopic experience. Cochr Database Syst Rev.

[32] Sidhu RS, Park J, Brydges R, MacRae HM, Dubrowski A (2007). Laboratory-based vascular anastomosis training: a randomized controlled trial evaluating the effects of bench model fidelity and level of training on skill acquisition. J Vasc Surg.

[33] Thomsen AS (2017). Operating room performance improves after proficiency-based virtual reality cataract surgery training. Ophthalmology.

[34] Thomsen AS, Smith P, Subhi Y, Cour M, Tang L, Saleh GM, Konge L (2017). High correlation between performance on a virtual-reality simulator and real-life cataract surgery. Acta Ophthalmol.

[35] Sturm LP, Windsor JA, Cosman PH, Cregan P, Hewett PJ, Maddern GJ (2008). A systematic review of skills transfer after surgical simulation training. Ann Surg.

[36] Cheng H, Clymer JW, Po-Han Chen B, Sadeghirad B, Ferko NC, Cameron CG, Hinoul P (2018). Prolonged operative duration is associated with complications: a systematic review and meta-analysis. J Surg Res.

[37] Vonlanthen R (2011). The impact of complications on costs of major surgical procedures: a cost analysis of 1200 patients. Ann Surg.

[38] Childers CP, Maggard-Gibbons M (2018). Understanding costs of care in the operating room. JAMA Surg.

[39] Hafford ML, Van Sickle KR, Willis RE, Wilson TD, Gugliuzza K, Brown KM, Scott DJ (2013). Ensuring competency: are fundamentals of laparoscopic surgery training and certification necessary for practicing surgeons and operating room personnel?. Surg Endosc.

[40] Surgeons SoAGaE (2018) Fundamentals of Laparoscopic Skills (FLS) Program. Accessed 28 September 2018 2018www.flsprogram.org

[41] Allen K, Blascovich J (1994). Effects of music on cardiovascular reactivity among surgeons. JAMA.

[42] Hawksworth C, Asbury AJ, Millar K (1997). Music in theatre: not so harmonious. A survey of attitudes to music played in the operating theatre. Anaesthesia.

[43] Hawksworth CR, Sivalingam P, Asbury AJ (1998). The effect of music on anaesthetists’ psychomotor performance. Anaesthesia.

[44] Way TJ, Long A, Weihing J, Ritchie R, Jones R, Bush M, Shinn JB (2013). Effect of noise on auditory processing in the operating room. J Am Coll Surg.

[45] Weldon SM, Korkiakangas T, Bezemer J, Kneebone R (2015). Music and communication in the operating theatre. J Adv Nurs.

[46] Graafland M, Schraagen JM, Boermeester MA, Bemelman WA, Schijven MP (2015). Training situational awareness to reduce surgical errors in the operating room. Br J Surg.

[47] Hull L, Arora S, Aggarwal R, Darzi A, Vincent C, Sevdalis N (2012). The impact of nontechnical skills on technical performance in surgery: a systematic review. J Am Coll Surg.

[48] He W, Ni S, Chen G, Jiang X, Zheng B (2014). The composition of surgical teams in the operating room and its impact on surgical team performance in China. Surg Endosc.

[49] Kurmann A, Keller S, Tschan-Semmer F, Seelandt J, Semmer NK, Candinas D, Beldi G (2014). Impact of team familiarity in the operating room on surgical complications. World J Surg.

[50] Mazzocco K (2009). Surgical team behaviors and patient outcomes. Am J Surg.

